# Supplementary material for: Modeling glioblastoma heterogeneity as a dynamic network of cell states
Source: Mol Syst Biol. 2021 Sep 16;17(9):e10105. doi: 10.15252/msb.202010105 (PMC8444284; doi:10.15252/msb.202010105)
Supplement: Supplementary file 6 — Source Data for Figure 5 [file MSB-17-e10105-s004.zip › Figure5A_sourcedata/GSEA_3017/nosigdb_stateA.GseaPreranked.1622795026485/VANGRONINGEN_MES.html]

Details for gene set VANGRONINGEN\_MES[GSEA]

|  || Dataset | state53017 |
| Phenotype | NoPhenotypeAvailable |
| Upregulated in class | na\_neg |
| GeneSet | VANGRONINGEN\_MES |
| Enrichment Score (ES) | -0.46668804 |
| Normalized Enrichment Score (NES) | -2.8294346 |
| Nominal p-value | 0.0 |
| FDR q-value | 0.0 |
| FWER p-Value | 0.0 |
Table: GSEA Results Summary

  

Fig 1: Enrichment plot: VANGRONINGEN\_MES      
 Profile of the Running ES Score & Positions of GeneSet Members on the Rank Ordered List

  

| PROBE | GENE SYMBOL | GENE\_TITLE | RANK IN GENE LIST | RANK METRIC SCORE | RUNNING ES | CORE ENRICHMENT || 1 | TM4SF1 |  |  | 1 | 1.087 | 0.0261 | No |
| 2 | VIM |  |  | 46 | 0.543 | -0.0089 | No |
| 3 | ADGRG6 |  |  | 86 | 0.467 | -0.0402 | No |
| 4 | CTSC |  |  | 96 | 0.456 | -0.0387 | No |
| 5 | RGS10 |  |  | 110 | 0.445 | -0.0419 | No |
| 6 | DNAJC1 |  |  | 151 | 0.407 | -0.0759 | No |
| 7 | TNC |  |  | 159 | 0.402 | -0.0735 | No |
| 8 | ABRACL |  |  | 182 | 0.388 | -0.0881 | No |
| 9 | SERPINE2 |  |  | 201 | 0.373 | -0.0986 | No |
| 10 | RAB13 |  |  | 220 | 0.366 | -0.1093 | No |
| 11 | UGDH |  |  | 224 | 0.363 | -0.1036 | No |
| 12 | PLS3 |  |  | 236 | 0.355 | -0.1068 | No |
| 13 | HOMER1 |  |  | 255 | 0.346 | -0.1180 | No |
| 14 | ITGB1 |  |  | 256 | 0.346 | -0.1094 | No |
| 15 | HSPB1 |  |  | 312 | 0.322 | -0.1620 | No |
| 16 | CREB3L2 |  |  | 348 | 0.310 | -0.1928 | No |
| 17 | P4HA1 |  |  | 411 | 0.290 | -0.2539 | No |
| 18 | NFIA |  |  | 413 | 0.289 | -0.2478 | No |
| 19 | ARMCX2 |  |  | 457 | 0.279 | -0.2882 | No |
| 20 | AMMECR1 |  |  | 481 | 0.274 | -0.3067 | No |
| 21 | MANF |  |  | 521 | 0.266 | -0.3431 | No |
| 22 | PXDN |  |  | 524 | 0.266 | -0.3387 | No |
| 23 | ZFP36L1 |  |  | 532 | 0.264 | -0.3398 | No |
| 24 | FZD2 |  |  | 551 | 0.259 | -0.3531 | No |
| 25 | SOX9 |  |  | 577 | 0.254 | -0.3744 | No |
| 26 | ITGAV |  |  | 599 | -0.250 | -0.3913 | No |
| 27 | NES |  |  | 603 | -0.252 | -0.3883 | No |
| 28 | SYPL1 |  |  | 608 | -0.255 | -0.3863 | No |
| 29 | NR3C1 |  |  | 610 | -0.256 | -0.3810 | No |
| 30 | FLNA |  |  | 636 | -0.264 | -0.4020 | No |
| 31 | SHROOM3 |  |  | 639 | -0.264 | -0.3976 | No |
| 32 | GNG12 |  |  | 640 | -0.265 | -0.3909 | No |
| 33 | PEA15 |  |  | 668 | -0.281 | -0.4137 | No |
| 34 | RHOJ |  |  | 682 | -0.293 | -0.4207 | No |
| 35 | EFEMP2 |  |  | 686 | -0.294 | -0.4166 | No |
| 36 | FZD7 |  |  | 721 | -0.316 | -0.4462 | No |
| 37 | EMP1 |  |  | 724 | -0.322 | -0.4404 | No |
| 38 | HES1 |  |  | 736 | -0.331 | -0.4443 | No |
| 39 | PON2 |  |  | 743 | -0.338 | -0.4424 | No |
| 40 | BOC |  |  | 766 | -0.359 | -0.4577 | Yes |
| 41 | DLX1 |  |  | 770 | -0.361 | -0.4520 | Yes |
| 42 | SPARC |  |  | 782 | -0.369 | -0.4549 | Yes |
| 43 | TPM1 |  |  | 784 | -0.369 | -0.4468 | Yes |
| 44 | SQSTM1 |  |  | 795 | -0.379 | -0.4484 | Yes |
| 45 | TRAM2 |  |  | 797 | -0.382 | -0.4399 | Yes |
| 46 | PHLDA3 |  |  | 798 | -0.382 | -0.4304 | Yes |
| 47 | NPC2 |  |  | 800 | -0.383 | -0.4219 | Yes |
| 48 | SEC14L1 |  |  | 807 | -0.388 | -0.4188 | Yes |
| 49 | APP |  |  | 812 | -0.394 | -0.4134 | Yes |
| 50 | MRC2 |  |  | 813 | -0.395 | -0.4035 | Yes |
| 51 | LTBP1 |  |  | 815 | -0.397 | -0.3947 | Yes |
| 52 | GJA1 |  |  | 823 | -0.405 | -0.3923 | Yes |
| 53 | PRRX1 |  |  | 829 | -0.413 | -0.3875 | Yes |
| 54 | CD59 |  |  | 831 | -0.422 | -0.3780 | Yes |
| 55 | FILIP1L |  |  | 835 | -0.428 | -0.3706 | Yes |
| 56 | PLOD2 |  |  | 838 | -0.431 | -0.3621 | Yes |
| 57 | SDCBP |  |  | 845 | -0.438 | -0.3578 | Yes |
| 58 | IFITM2 |  |  | 864 | -0.460 | -0.3661 | Yes |
| 59 | ANXA2 |  |  | 872 | -0.482 | -0.3618 | Yes |
| 60 | ERBIN |  |  | 876 | -0.490 | -0.3529 | Yes |
| 61 | PDGFC |  |  | 883 | -0.515 | -0.3466 | Yes |
| 62 | CALD1 |  |  | 889 | -0.527 | -0.3390 | Yes |
| 63 | GPC6 |  |  | 896 | -0.540 | -0.3321 | Yes |
| 64 | A2M |  |  | 898 | -0.545 | -0.3196 | Yes |
| 65 | PLK2 |  |  | 901 | -0.551 | -0.3080 | Yes |
| 66 | SDC2 |  |  | 908 | -0.568 | -0.3004 | Yes |
| 67 | DKK3 |  |  | 912 | -0.579 | -0.2893 | Yes |
| 68 | RAB31 |  |  | 916 | -0.586 | -0.2779 | Yes |
| 69 | ID1 |  |  | 931 | -0.651 | -0.2771 | Yes |
| 70 | LRRC17 |  |  | 949 | -0.769 | -0.2766 | Yes |
| 71 | GDF15 |  |  | 952 | -0.790 | -0.2591 | Yes |
| 72 | MMP2 |  |  | 953 | -0.829 | -0.2384 | Yes |
| 73 | IFITM3 |  |  | 954 | -0.830 | -0.2177 | Yes |
| 74 | CD44 |  |  | 957 | -0.851 | -0.1986 | Yes |
| 75 | COL5A2 |  |  | 958 | -0.856 | -0.1772 | Yes |
| 76 | CNN3 |  |  | 961 | -0.883 | -0.1573 | Yes |
| 77 | ANXA1 |  |  | 972 | -1.018 | -0.1429 | Yes |
| 78 | CYR61 |  |  | 978 | -1.296 | -0.1161 | Yes |
| 79 | ID3 |  |  | 981 | -1.505 | -0.0807 | Yes |
| 80 | IGFBP5 |  |  | 983 | -1.595 | -0.0419 | Yes |
| 81 | SPARCL1 |  |  | 984 | -1.810 | 0.0033 | Yes |
Table: GSEA details [plain text format]

  

Fig 2: VANGRONINGEN\_MES: Random ES distribution      
 Gene set null distribution of ES for **VANGRONINGEN\_MES**
